# Overexpression of *OsDUF6* increases salt stress tolerance in rice

**DOI:** 10.1186/s12870-024-04921-z

**Published:** 2024-03-26

**Authors:** Guangming Ma, Yong Zhang, Xiangyang Li

**Affiliations:** National Key Laboratory of Green Pesticide, Key Laboratory of Green Pesticides and Agricultural Bioengineering, Ministry of Education, Guizhou University, Guiyang, 550025 China

**Keywords:** Rice, *OsDUF6*, Salt stress, Functional analysis, Overexpression

## Abstract

**Background:**

Soil salinity is one of the primary environmental stresses faced in rice production. When plants are exposed to salt stress, a series of cellular balances will be disrupted. Dufulin is an immune-induced antiviral agent used in plants. The *DUF* gene family influences plant response to abiotic stress, and the functional role of *OsDUF6*(ABA98726.1) in rice response to salt stress is being investigated here.

**Results:**

Based on the transcriptome analysis of Dufulin treatment in inducing salt tolerance in rice, we selected the OsDUF6 protein located on the cell membrane and studied its molecular function by overexpressing *OsDUF6*. Salt-induced decreases in root, stem, and leaf length and increased leaf yellowing rate and Na^+^ concentration in the wild-type plant were mitigated in the overexpressed lines. *OsDUF6* overexpression increased the enzymatic antioxidant activities of superoxide dismutase, peroxidase, catalase, and phenylalanine ammonia-lyase. *OsDUF6* also played a positive role in Na^+^ transport as reflected by the increased growth of a salt-sensitive yeast mutant complemented with *OsDUF6* in the presence of salt stress. In addition, Reverse transcription quantitative PCR analysis confirmed that the overexpression of *OsDUF6* significantly changed the expression level of other genes related to growth and stress tolerance.

**Conclusions:**

Combined with previously published data, our results supported the observation that *OsDUF6* is an important functional factor in Dufulin-induced promotion of salt stress tolerance in rice.

**Supplementary Information:**

The online version contains supplementary material available at 10.1186/s12870-024-04921-z.

## Introduction

One of the major abiotic stresses encountered by food crops during growth is increased soil salinity [1–3]. More than 20 % of the world’s agricultural land is affected by high salinity, a figure which is expected to rise in the future [4]. When food crops are subjected to salt stress, the plants respond at the morphological, cellular, and molecular levels [5]. When encountering salt stress, the homeostasis between reactive oxygen species (ROS) production and scavenging will be broken, resulting in excessive accumulation of ROS, which will damage the original biological functions of the plant to a certain extent and hinder the normal physiological processes of the plant [6, 7]. Plant morphological changes under high salt conditions included impaired root establishment, increased occurrence of leaf rolling and yellowing, decreased thousand-seed weight, and decreased number of spikelets per ear, the latter ultimately resulting in decreased harvest index and grain yield [8, 9].

Rice (*Oryza sativa* L.) is the main food crop for much of the world’s population and is also one of the most important staple foods in daily life [10]. With climatic changes observed in recent years, rice needs to respond to biological and abiotic stresses. The use of pesticides and fungicides has reduced the importance of biological stresses in rice production. However, rice also needs to respond to several abiotic stresses. Among these, salt stress is an important focus of current research, and it has been found that rice is more sensitive to drought and salt stress than other cereals, such as barley, wheat, and rye [11, 12], making rice an urgent target for studies into mechanisms of salt tolerance. At present, people pay more attention to healthy eating, while the quality of rice as a staple food is also a key target [13, 14]. Additionally, achieving the best of both worlds – increasing the yield of rice while improving its quality – is a goal desired by many. Despite this, it has become a major public issue to improve rice production by promoting rice tolerance to salt stress [15, 16].

Based on the transcriptomic data from rice plants exposed to salt stress after being sprayed with the plant antiviral agent Dufulin, which has been shown to induce salt stress tolerance in rice, we found that *OsDUF6*(ABA98726.1) exhibited significantly altered expression among the differentially expressed genes (DEGs) [17]. Studying salt stress in response to spraying with a disease-resistance inducer, such as Dufulin, is in line with the current environment of how to tolerate abiotic stress after preventing biotic stress. Analysis of the molecular function of *OsDUF6* will allow us to confirm whether a gene conferring tolerance to salt stress, the expression of which changes in response to treatment with a chemical, Dufulin, which induces salt tolerance, is an important gene with respect to improving response to abiotic stresses. Domain of Unknown Function (DUF) proteins are a large set of uncharacterized proteins with conserved amino acid sequences but unknown functions [18]. OsDUF6 is a member of the DUF protein family. According to version 35.0 of the Pfam database, the DUF family accounts for 24% of all plant protein families [19]. Despite not being essential for plant survival, research has shown that the DUF family plays a crucial role in physiological processes, including plant growth, development, and responses to biotic and abiotic stresses [20–22].

Therefore, based on an in-depth investigation of transcriptomic data in response to chemical compounds that promote salt tolerance in rice, this current study investigated the molecular function of *OsDUF6* and its positive impact on rice salt tolerance after overexpression. These studies can provide new insights into the molecular function of rice salt inducers, identify new salt-tolerance genes for exploitation in molecular breeding, and enrich our understanding of the rice salt stress signaling network, including exploration of the DUF family.

## Materials and methods

### Bioinformatic analysis of the OsDUF6 gene

The sequences of the OsDUF6 protein and its homologs were obtained from the NCBI. In the ExPASy2 website (http://au.expasy.org/tools/protparam.html), physical and chemical parameters were predicted for preliminary OsDUF6 proteins [23]. Based on this preliminary information, the subcellular localization (http://www.csbio.sjtu.edu.cn/bioinf/Cell-PLoc-2/), the protein secondary structure (http://bioinf.cs.ucl.ac.uk/psipred/), interaction protein database (https://cn.string-db.org/), tertiary structure model (https://www.uniprot.org/), and transmembrane signal peptide (https://www.dtu.dk/english) parameters of OsDUF6 were predicted and analyzed online [24–29]. Based on the *OsDUF6* gene sequence, a search was carried out for homologous sequences among multiple species in NCBI; any sequence identified was downloaded and MEGA software was used to carry out multiple sequence alignments, and the neighbor-joining method was used to construct a phylogenetic tree [30].

### General subcellular localization experiment

The coding sequence of *OsDUF6* (LOC_Os12g33300) was cloned into thepCam35S-GFP vector. The recombinant plasmid was then used to transform *Agrobacterium tumefaciens* strain GV3101 in its competent state and the bacteria were cultured in a 28°C incubator for 2–3 d. After screening a single colony to confirm its transformation-positive status, the culture was multiplied in a liquid Luria-Bertani(LB) medium. After centrifugation, the bacterial pellet was resuspended and, after dark incubation for 3 h, was inoculated into leaves of 2-week-old *Nicotiana benthamiana* seedlings of uniform growth form and development. Three days later, the injected leaves were cut into 2 mm × 2 mm squares and placed on slides, with the fluorescence from the green fluorescent protein (GFP) being observed under a confocal laser scanning microscope (Zeiss, Oberkochen, Germany).

### Sources and cultivation of transgenic overexpressed rice

The *pBWA (V) HS-OsDUF6* plasmid was constructed, and 1 μL plasmid was added to 50 μL strain EHA105 *Agrobacterium* competent cells for transformation. Then, *Agrobacterium* infection co-culture, callus screening and induce rooting [31, 32]. The transgenic rice seedlings were then transferred to plastic pots containing a soil/perlite mixture and grown in a growth chamber at 26 °C and 72 % relative humidity under long-day 16 h/8 h (day/night) photoperiod conditions. Using the cetyltrimethylammonium bromide (CTAB) method to extract rice genomic DNA from the rice seedlings, PCR was then used to detect positive plants.

### Salt stress treatment of plant material

Full and intact seeds of homozygous overexpressed and wild-type rice (‘Nipponbare’) of similar size were selected and germinated for 3–5 d. After germination, they were transplanted into plastic pots of a soil/perlite mixture in the greenhouse [26 °C and 72 % relative humidity under 16 h/8 h (day/night) photoperiod]. After 15 d growth, 200 mM NaCl were applied to wild-type and transgenic rice seedlings to induce salt stress and were photographed and sampled after a further sixth days.

### Rice sample collection and total RNA extraction

The roots, stems, and leaves of the seedlings of overexpressed transgenic and wild-type rice cv. Nipponbare were collected after six days of salt stress. They were snap-frozen in liquid nitrogen and stored at −80 °C. Total RNA was extracted from the preserved sample tissues of the two lines, following the instructions of the manufacturer of the RNAprep Pure Plant Kit (Tiangen, Beijing, China). The RNA concentration was measured with a NanoPhotometer (Implen, Munich, Germany).

### Physiological indices

The phenotypes of transgenic and wild-type rice ‘Nipponbare’ grown in a greenhouse with or without six days exposure to salt stress were observed and recorded, with root, stem, and leaf lengths being measured to evaluate any growth differences. From a total of ten leaves, the percentage of yellowed leaves was determined to calculate the yellowing rate. The sodium (Na^+)^ concentrations of rice plants were measured by flame spectrometry.

For enzyme activity assays, The weight of rice tissue was accurately weighed, and 9 times the volume of normal saline was added according to the ratio of weight (g) to volume (mL) 1 : 9. Prepare a 10% tissue homogenate under ice water bath conditions. The homogenate was centrifuged at 2,500 rpm for 10 minutes and the supernatant was retained. Then, according to the instructions provided by the manufacturers of superoxide dismutase (SOD), peroxidase (POD), and catalase (CAT) assay kits (Jiancheng Bioengineering Institute, Nanjing, China), the tissue homogenate was added to the specific reagents to carry out the assay reaction and the absorbance values at wavelengths of 450 nm, 420 nm, and 405 nm, respectively, were measured using a Synergy microplate reader (Agilent, California, USA) , and calculate the activity values of the three enzymes were calculated using the equations given in reference [17].

For phenylalanine ammonia-lyase (PAL) enzyme activity, the appropriate PAL kit was used, following the instructions provided by the kit manufacturer (Jiancheng Bioengineering Institute, Nanjing, China). The extraction buffer was added to the tissue at a ratio of 9 mL buffer per g tissue weight. The mixture was homogenized under ice water bath conditions, centrifuged at 10,000 rpm for 10 minutes, and the supernatant was collected. The substrate reagents were added to the crude enzyme solution, incubate at 30 °C for 30 minutes, and then add the colorimetric reagent was added. The solution was mixed well, allowed to stand for 10 minutes, and the absorbance value at 290 nm was determined with a Synergy microplate reader (Agilent, California, USA), allowing the enzyme activity value to be calculated using the appropriate equation [17].

### Reverse transcription quantitative PCR (RT-qPCR) analysis

The total RNA, extracted as described in section " Rice sample collection and total RNA extraction", was converted into cDNA using the StarScript III All-in-One RT Mix with gDNA Kit (Genstar, Beijing China), following the manufacturer’s instructions and diluted with ddH_2_O. In the 20 μL reaction volume, 2 × RealStar Fast SYBR qPCR Mix (Genstar, China) was used for premixing, and diethylpyrocarbonate (DEPC)-treated H_2_O was used to adjust the volume. The reaction was carried out in the LightCycler 96 RT-qPCR assay system (Roche, Basel, Switzerland) and the reaction was completed according to the dissolution curve automatically set by the instrument. The relative gene expression rate between the overexpression lines and the wild-type lines was compared using the 2^−△△Ct^ method [33], and three biological replicates were conducted for each sample. All quantitative PCR primers are listed in Table S1 and were synthesized by Beijing Tsingke Biology (Beijing, China).

### Confirmation of yeast functional complementation

We used a salt-sensitive *Saccharomyces cerevisiae* mutant *AXT3K* that lacks the main plasma membrane Na^+^ transporter [34]. The full-length sequence of *OsDUF6* was cloned into the PYES2 yeast expression vector. First, the *AXT3K* strain, stored at –80°C, was activated, and then, according to the manufacturer's instructions, the yeast transformation kit (Coolaber, Beijing, China) was used to convert the strain into the receptive state. Then, the *OsDUF6*-containing recombinant plasmid was introduced into the prepared receptive *AXT3K* by the PEG/LiAc method, and the transformed yeast, after centrifugation, was spread onto SD/-Ura solid medium, with the plate being cultured, in an inverted orientation, in a 30 °C incubator for 3–4 d to achieve yeast colonies of the appropriate size. After a single colony was selected and confirmed to be transformation-positive, the transformed yeast was suspended in ddH_2_O and diluted with ddH_2_O to form the concentration gradient of 10-fold serial dilutions. Approximately 3 μL of the yeast suspension was applied onto the Arg phosphate (AP) solid medium plate containing 0, 25, 50, or 75 mM NaCl, incubated upside down at 30 °C to grow for 3–4 d, and the yeast growth was measured and recorded.

### Data analysis

All data were analyzed by analysis of variance (ANOVA) and Tukey’s HSD test was selected to identify statistically significant (*P* < 0.05 and *P* < 0.01)) differences in measured parameters between the wild-type and overexpression lines. Three independent biological replicates were used for each measurement. Graphs were generated using Prism software (v 9.0) (GraphPad Software, Boston, MA, USA)**.**

## Results

### Functional prediction and analysis of the OsDUF6 protein

**Fig. 1:**
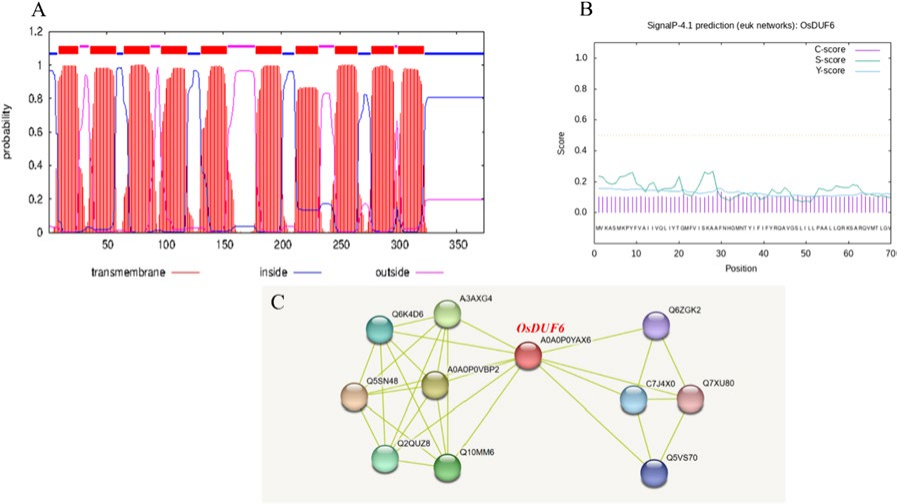
Functional prediction and analysis. **A** Prediction of OsDUF6 protein transmembrane region; **B** Secretory signal peptide; **C** Prediction of OsDUF6 interacting protein regulatory network

The molecular weight and isoelectric point (pI) of OsDUF6 were 41.46 kDa and 8.87, respectively, and the number of amino acids encoded was 373. Through the prediction of its transmembrane domain and signal peptide, the results showed that the deduced protein had ten transmembrane domains, so that it was a transmembrane protein, but lacked a signal peptide (Fig. 1A, B). The subcellular localization of the protein was predicted online and indicated to be in the cell membrane (Fig. S1). The secondary structure of OsDUF6 was predicted by PSIPRED. It can be seen from the prediction results that helixes and coils constituted almost the entire secondary structure of OsDUF6, with strands encoding only two amino acids, so that the secondary structure is relatively stable (Fig. S2). Then, on the basis of the secondary structure, we predicted the tertiary structure of OsDUF6, with the results showing that the AlphaFold produced a very high per-residue confidence score (pLDDT) >90, so that this tertiary structure could be applied to experimental research to a certain extent (Fig. S3). By comparing and screening with the STRING database, based on the rice variety studied in this experiment, the *Oryza sativa Japonica* Group was selected to predict the proteins interacting with OsDUF6 in the database. In Fig. 1C, A0A0P0YAX6 is our target protein, and the intricate lines represent the interaction network of OsDUF6; this interaction network is clearly divided into two parts. However, according to the meaning of the line representation, we found that the prediction results are based on the mined text, and the proteins interacting with OsDUF6 are not based on accurate data or experimental results, which will require us to determine the actual interacting proteins based on experimental results (Fig. 1C).

**Fig. 2:**
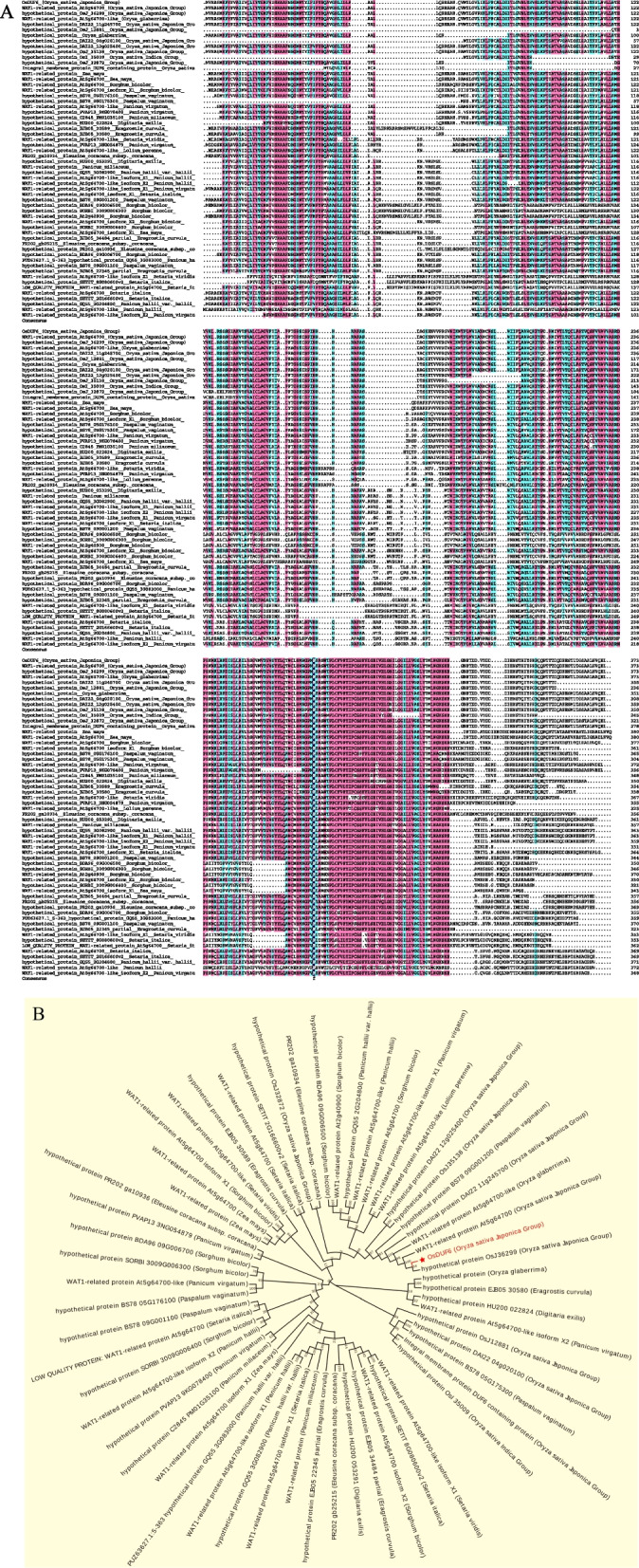
Homology sequence alignment and phylogenetic tree analysis of *OsDUF6*. **A**, Homology sequence alignment; **B**, Phylogenetic tree analysis)

The homologous sequence of the OsDUF6 protein sequence was studied using NCBI technology. Then, MEGA6.0 software was used to achieve multiple sequence comparisons, and the amino acid sequence obtained after comparison was used to construct an evolutionary phylogenetic tree. The results showed that OsDUF6 had a high degree of genetic relationship with the WAT1-related protein At5g64700 (*O. sativa japonica* Group) and hydroxy protein OsJ36299 (*O. sativa japonica* Group), but it had a distant genetic relationship with the hydroxy protein BS78-09G001100 (*Paspalum vaginatum*) and the hydroxy protein EJB05-22345 partial (*Eragrostis curvula*) (Fig. 2A, B).

### OsDUF6 encodes a membrane-localized protein

**Fig. 3:**
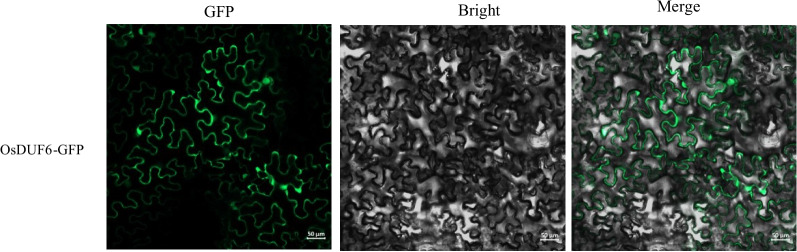
General subcellular localization experiment of OsDUF6 protein. (GFP: Green Fluorescent Protein)

In order to detect the general subcellular localization of the OsDUF6 protein, the coding sequence (CDS) region of *OsDUF6* was cloned and the expression vector of the *OsDUF6-GFP* (green fluorescent protein) fusion protein was constructed and was used to transform *Agrobacterium*, which was cultured, incubated, and injected into tobacco. Finally, the subcellular localization was preliminarily determined by laser scanning confocal microscopy to detect the fluorescence signal. As shown in Fig. 3, the fluorescence of the 35S::*OsDUF6-GFP* construct was detected in the cell membrane. Comparison of the prediction results and the subcellular localization results showed that they were similar, so that the *OsDUF6* gene was determined to encode a membrane-localized protein.

### Functional analysis of OsDUF6 in yeast mutants under NaCl stress

**Fig. 4:**
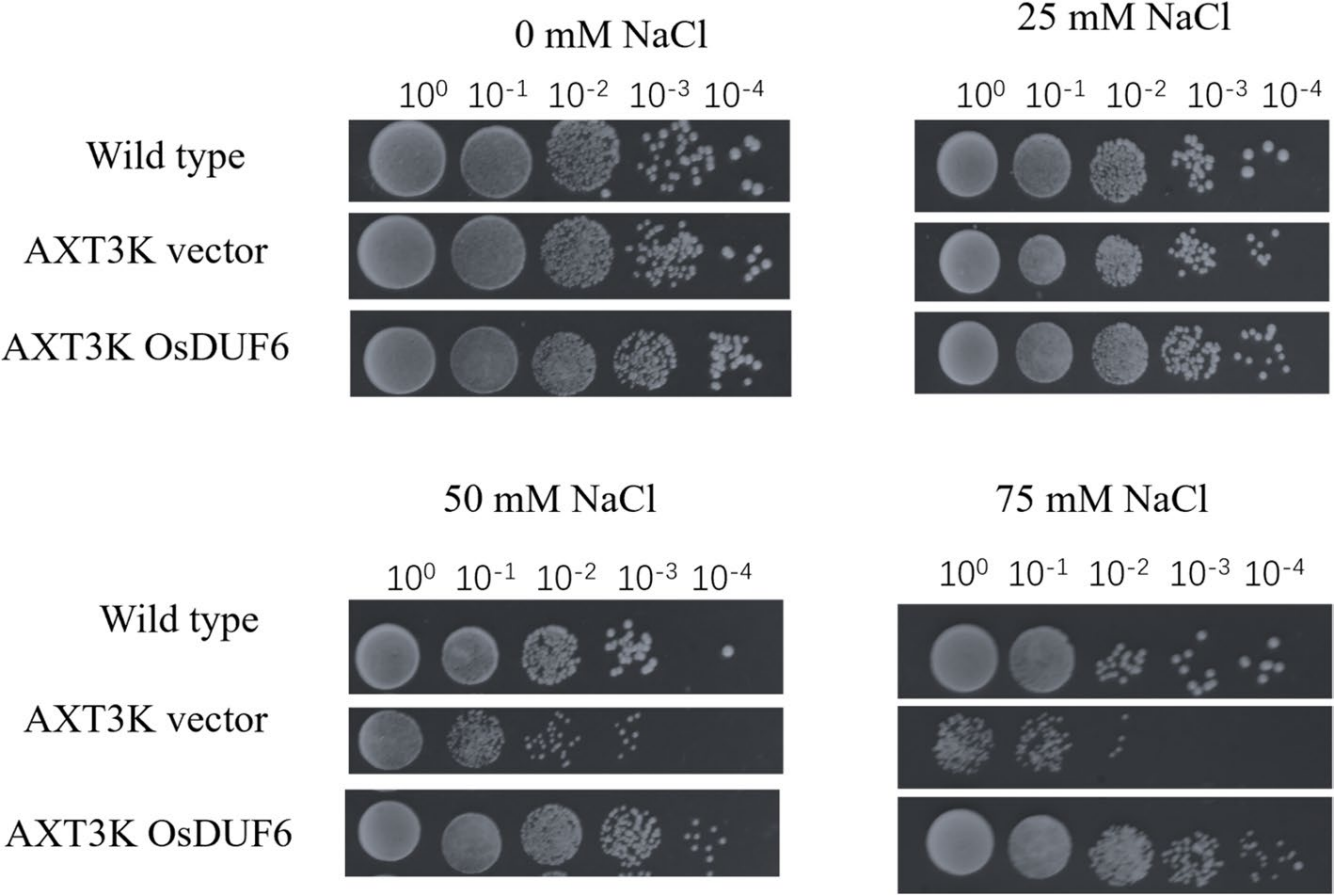
*OsDUF6* gene promotes the growth of yeast mutant AXT3K under salt stress

In order to test the function of *OsDUF6* in salt tolerance, *OsDUF6* was cloned into yeast expression vector pYES2, and then transformed into *S. cerevisiae* mutant *AXT3K* (ena1–4:: HIS3,nha1:: LEU2, nhx1:: KanMX), which is very sensitive to Na^+^ stress. For each transformed yeast strain, colony growth responded inversely to a gradient of NaCl concentrations on AP medium. The results showed that the yeast mutant carrying the *OsDUF6* gene still grew at the highest NaCl concentration on the AP medium, whereas the control mutant group barely grew (Fig. 4). This growth of the complemented yeast mutant confirmed the functional salt tolerance encoded by the *OsDUF6* gene.

### Overexpression of OsDUF6 promotes salt tolerance in rice

**Fig. 5:**
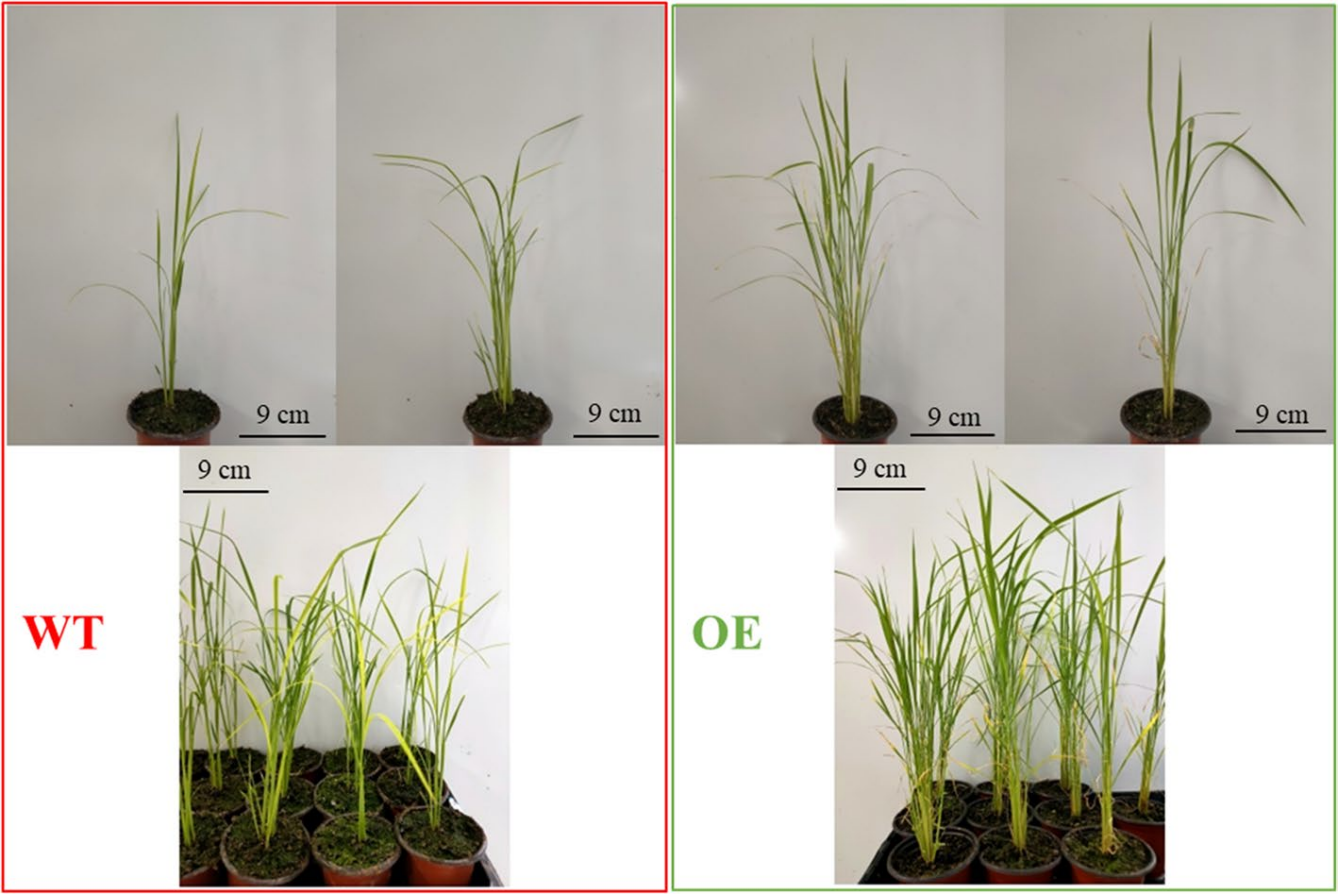
Growth characteristics of rice in response to salt stress. (left, wild-type; right, OE)

In order to clarify the function of *OsDUF6* transgenic rice in terms of salt stress response, seeds of the transgenic rice lines and the corresponding wild-type plant were grown in a greenhouse, and 200 mM NaCl was added at the soil matrix at a specified time to achieve salt stress treatment. Under normal, non-stress conditions, there was no significant phenotypic difference between transgenic lines and the wild-type strain. After the sixth day of salt treatment, according to phenotypic observations, all rice plants (transgenic lines and wild type) were damaged, although to varying extents (Fig. 5).

**Fig. 6:**
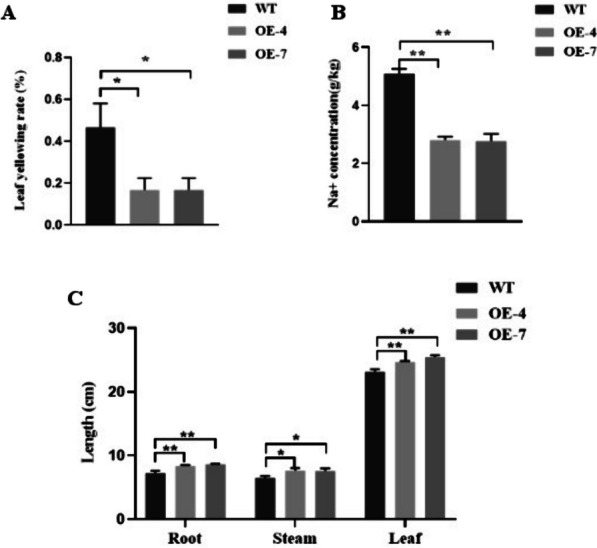
Physiological indicators of wild type (WT) and OsDUF6-overexpressed (OE) lines under salt stress. **A** Leaf yellow rate; **B** Na^+^ content; **C** Length of root, stem and leaf. [mean values displayed in each bar followed by different letters significantly differ according to Student’s t-test (*P < 0.05, **P < 0.01). Vertical bars indicate SD (*n* = 3)]

Na^+^ homeostasis is an important characteristic of plant salt tolerance. Therefore, we determined the concentration of Na^+^ in the roots and leaves of wild-type and overexpressed lines. Compared with the Na^+^ concentration of the wild-type plants, the accumulation of Na^+^ in the overexpression lines was significantly lower, which indicates that the stress tolerant mechanism was due, at least in part, to the transgenic plants being more efficient at transporting Na^+^ into/out of the plant cells (Fig. 6A). The area of yellowed tissue of leaves of the transgenic plants was smaller than that of the wild-type plants; furthermore, the length of the roots, leaves, and stems of both genotypes were significantly reduced under salt stress (compared with the corresponding non-stressed controls), but the decrease in transgenic plant size was much smaller than that of the wild-type plants (Fig. 6B, C).

### Overexpression of OsDUF6 increases the activity of antioxidant enzymes under salt stress

**Fig. 7:**
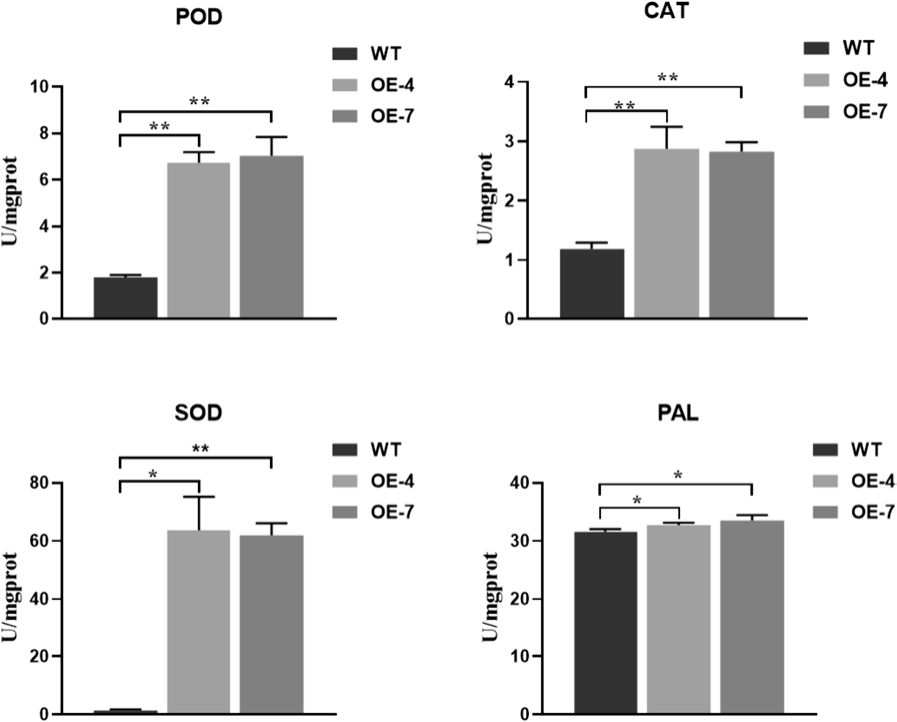
Activities of antioxidant ROS-scavenging enzymes in rice under salt stress. [mean values displayed in each bar followed by different letters significantly differ according to Student’s t-test (*P < 0.05, **P < 0.01). Vertical bars indicate SD (*n* = 3)]

In order to detect the level of ROS scavenging in overexpressed lines and wild-type plants under salt stress conditions, we measured the activities of the main direct antioxidant enzymes, namely SOD, CAT, and POD, and an indirect antioxidant enzyme, PAL, in the overexpressed and wild-type lines. After assay, it was found that, in response to salt treatment, the increases in SOD, PAL, CAT, and POD activities in overexpression lines were higher than those in the wild-type lines (Fig. 7), with the increase in the three direct antioxidant enzymes being much higher than that of the indirect antioxidant enzyme, PAL. These results suggest that *OsDUF6* overexpression lines can increase the activity of four antioxidant enzymes SOD, PAL, CAT and POD when rice is exposed to salt stress, so that the accumulation of ROS in rice under salt stress would be reduced to a certain extent, compared with wild-type lines, thereby alleviating a series of adverse oxidative stress effects caused by ROS accumulation in rice and promoting rice tolerance to salt stress.

### Overexpression of OsDUF6 changed the expression of related genes under salt stress

**Fig. 8:**
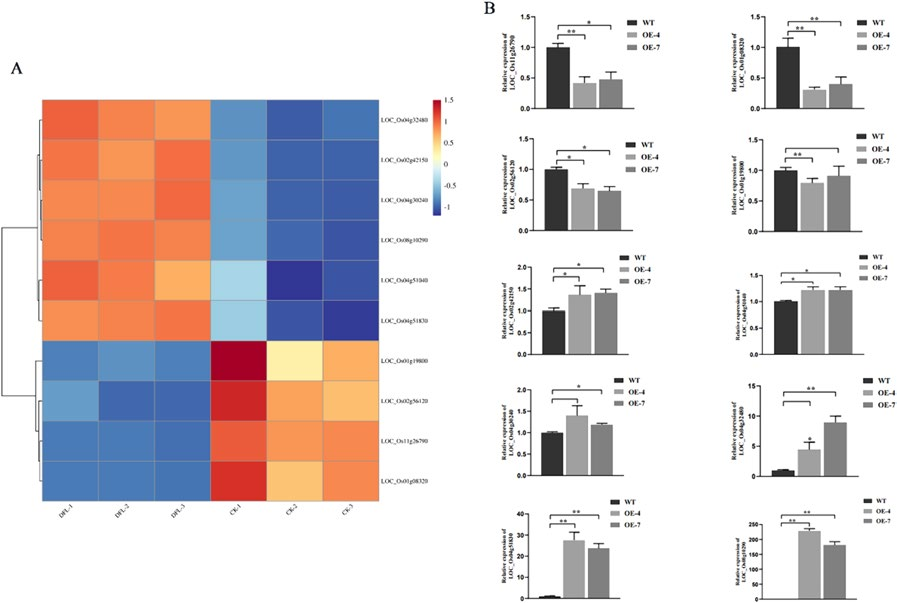
Differentially expressed genes (DEGs) associated with salt stress treatment. **A** Cluster heat map of DEGs. Red indicates up-regulated genes, and blue indicates down-regulated genes. Ten selected gene names were marked. (DFL, Dufulin-treated group; CK, control group); (**B**) RT-qPCR validation. Ten genes related to growth and stress resistance were verified. [mean values displayed in each bar followed by different letters significantly differ according to Student’s t-test (*P < 0.05, **P < 0.01). Vertical bars indicate SD (*n* = 3)]

Our previous research had shown that, when rice was subjected to salt stress after spraying with Dufulin, *OsDUF6* expression in the rice transcriptome was found to be upregulated, with the relative expression of some other genes also changing markedly [17]. Therefore, we selected a number of these differentially expressed genes which were putatively related to growth and stress tolerance and the expression of these genes was presented as a heatmap, based on the previous transcriptome data (Fig. 8A). Based on these data, we conducted RT-qPCR to confirm the changes in expression level of these genes in the leaves of transgenic and wild-type plants. The results confirmed that the dynamic changes in expression of these genes were consistent with those from the previous transcriptome data and were associated with increased plant salt tolerance (Fig. 8B) [17]. The expression levels of *LOC_Os11g26790* (dehydrin), *LOC_Os01g08320* (OsIAA1 - Auxin-responsive Aux/IAA gene family member), *LOC_Os02g56120* (OsIAA9 - Auxin-responsive Aux/IAA gene family member) and *LOC_Os01g19800* (zinc finger, C3HC4 type) were down-regulated in the OE group, the *LOC_Os08g10290* (SHR5-receptor-like kinase), *LOC_Os02g42150* (OsWAK14 - OsWAK receptor-like protein kinase), *LOC_Os04g51040* (OsWAK50 - OsWAK receptor-like protein kinase), *LOC_Os04g30240* (OsWAK60 - OsWAK receptor-like protein kinase), *LOC_Os04g32480* (zinc-finger protein), *LOC_Os04g51830* (OsHKT1;4 - Na^+^ transporter) were up-regulated in the OE group. Therefore, these results indicate that overexpression of *OsDUF6* changed the expression level of these genes to a certain extent, which means that *OsDUF6* expression is closely related to that of these genes. In summary, according to the results of RT-qPCR, in the presence of salt stress, overexpression of *OsDUF6* has a positive effect on rice growth and expression of stress-related genes via an effect on Na^+^ transport, which further explains its role in promoting salt stress tolerance in rice.

## Discussion

Salinity is a major abiotic stress factor that seriously affects crop growth and yield, causing significant economic losses and threatening global food security [35, 36]. At present, 23.02 billion hectares of farmland worldwide can be used for food production. However, due to inappropriate crop irrigation methods (leading to salinization) and excessive fertilizer usage, as well as natural effects, such as sea level rise, resulting in salt intrusion into coastal areas, some 2 % of farmland is affected by salt, and the proportion of impacts on agricultural development is also increasing year by year [37]. Rice is a very important cereal crop that feeds half of the world's population and is particularly sensitive to salt stress [38]. There have been many studies on salt-tolerant transgenic rice, most of which were based on research into homologous species and gene families to identify the genes that change the salt response of rice [39–41]. Prior to the current study, the *OsDUF6* gene had no known function.

Several *DUF* family genes related to salt stress response have been reported. Overexpression of soybean *GmCBSDUF3*, encoding DUF21, can enhance the tolerance of *Arabidopsis thaliana* to both drought and salt stress [42]. Overexpression of *AhDGR2* (encoding a protein containing a DUF642 domain) in ‘Nipponbare’ resulted in increased sensitivity to NaCl treatment [43], *OsSIDP366* (a gene containing DUF1644) positively regulates the response of rice to drought and salt stress [44]. In the present study, based on the transcriptome analysis of rice sprayed with Dufulin and then subjected to salt stress [17], we studied the function of the *OsDUF6* gene, transgenic plants were generated to overexpress the *OsDUF6* gene, and their molecular functions were studied through a series of indicators. Research has shown that a high concentration of Na^+^ enters the body of plants under salt stress, inducing the accelerated accumulation of ROS [45, 46]. When a high concentration of ROS is generated, it will cause oxidative damage to the membranes; in serious cases, it can disrupt cellular metabolism, leading to the inhibition of normal growth and development of the plants [47, 48]. Through a subcellular protein localization test and software prediction, it was preliminarily determined that the OsDUF6 protein is located on the cell membrane (Fig. 3 and Fig. S1). Therefore, overexpression of *OsDUF6* will directly and positively affect the salt tolerance of rice by mediating Na^+^ transport through the membrane, a finding of great significance in the study of salt tolerance of rice.

When plants are exposed to salt stress, ROS will be generated, which will cause plants to be unable to eliminate them in time, resulting in ROS accumulation in cells and oxidative stress damage to particular components of the cells [49, 50]. Studies have also demonstrated that ROS accumulation depends largely on the balance between ROS production and concurrent ROS clearance by scavenging or quenching [51, 52]. ROS-scavenging enzymes play an important role in this process, with the activities of ROS-scavenging enzymes, such as SOD, CAT, and POD, affecting the ROS levels in stressed plants [53, 54].

Three research groups have previously reported that overexpressing genes encoding human leukocyte antigen-B associated transcript 1 (OsBAT1), late-embryogenesis-abundant (LEA) proteins (e.g., OsLEA1a), and plant ferritoxin-like protein (PFLP) in rice could enhance salt stress tolerance [55–57]. Overexpression of each of these genes could promote the production of ROS-scavenging enzymes, which helps to maintain the dynamic balance of ROS during salt stress. Therefore, the improved salt tolerance exhibited by such transgenic lines may be related to an increase in ROS-scavenging capacity during salt stress. In order to further clarify the effect of *OsDUF6* induction in response to salt stress in rice, we compared the activity of ROS scavenging enzymes in transgenic lines and wild-type plants. It was found that the activities of the ROS-scavenging enzymes CAT, POD, PAL, and SOD increased significantly in the *OsDUF6*-overexpressed transgenic lines (Fig. 7), compared with the corresponding wild-type line. In conclusion, the increased ROS-scavenging enzyme activity in the *OsDUF6-*overexpressed lines infers protection of the membrane system, mediation of the transport and prevention of the accumulation of Na^+^, and hence alleviation of the osmotic and oxidative stress damage, promoting the ability of the transgenic plants to tolerate salt stress.

Overexpression of *OsDUF6* stimulates dynamic changes in related genes when plants are exposed to salt stress. According to previous reports, *OsIAA1* plays a crucial role in crosstalk between auxin and brassinosteroid signaling pathways and hence in plant morphogenesis, whereas expression of *OsIAA9* is induced by various hormones and abiotic stresses, inhibiting auxin-regulated root growth [58, 59]. In the present study, decreased expression of OsIAA1 and OsIAA9 was observed after overexpression of *OsDUF6*, indicating that the overexpression of *OsDUF6* suppresses the expression of *OsIAA1* and *OsIAA9*. Furthermore, in response to salt stress, *OsDUF6*-overexpressing plants promote rice growth. Researchers have demonstrated that C3HC4-type zinc-finger (RING-finger) proteins represent one of the largest transcription factor families in the plant kingdom and play crucial roles in various plant processes such as regulating growth and development, signaling networks, and responses to abiotic stresses [60]. The C3HC4-type zinc-finger gene tested in our study was upregulated by *OsDUF6*, promoting its expression and enhancing rice growth and development under salt stress. Additionally, overexpression of *OsDUF6* impacts the expression of receptor-like kinases, known as receptor-like protein kinases (RLKs), a large protein family involved in regulating plant growth and development as well as responses to various stresses [61]. As is well known, RLKs play a crucial role in disease resistance. For example, OsWAK14, OsWAK91, and OsWAK92 confer resistance to rice blast disease [61], and a novel plant receptor kinase named SHR5, which interacts with endophytic nitrogen-fixing bacteria, has been identified [62]. Our measurements of the expression levels of genes encoding SHR5-receptor-like kinase, OsWAK14-OsWAK receptor-like protein kinase, OsWAK50-OsWAK receptor-like protein kinase, and OsWAK60-OsWAK receptor-like protein kinase revealed an increase in plants overexpressing *OsDUF6* in the presence of salt stress. This study lays the foundation for understanding the relationship between the DUF family and receptor-like kinases, as well as the role of receptor-like kinases in abiotic stress responses.

In addition, as part of an attempt to identify the function of *OsDUF6*, the gene was introduced into yeast mutant *AXT3K*, and characterization of the complemented line confirmed the role of the gene in maintaining monovalent cation homeostasis and salt stress tolerance by restoring the Na^+^ transport and tolerance of the *AXT3K* mutant. It was confirmed that the molecular functions of *OsDUF6* included increased tolerance to salt stress.

## Conclusions

This study is based on the promotion of salt tolerance in rice by Dufulin and the identification, by screening, of increased expression of the DUF gene family member *OsDUF6*. Through homologous sequence alignment and phylogenetic tree construction, the results show that OsDUF6 has the highest homology with the WAT1-related protein At5g64700 in the *O. sativa japonica* Group. Functional prediction analysis indicates that it is a protein without a signal peptide but with a transmembrane structure, and subcellular localization predictions and experiments further confirm its localization on the cell membrane. To verify its function, the overexpression of *OsDUF6* was preliminarily determined to promote salt tolerance in rice based on plant phenotype, chlorosis rate, and enzyme activity assays. Further verification through heatmap analysis established the association between Dufulin and the promotion of salt tolerance in rice. Using RT-qPCR, it was determined that overexpression of *OsDUF6* stimulates dynamic changes in related auxin and receptor-like kinase genes when exposed to salt stress, thereby altering salt tolerance. Finally, to explain the molecular function of these findings, complementation verification of salt tolerance was performed using the defective yeast mutant, *AXT3K*. In summary, OsDUF6 is a positive regulatory factor in response to salt stress in rice. These discoveries provide clues for exploring the function of the *DUF* family genes and lay the foundation for elucidating the biological functions of *OsDUF6*.

### Supplementary Information

**Supplementary Material 1.** 

## Data Availability

All of the datasets are included within the article and its additional files.

## Abbreviations

OE: Overexpression
SOD: Superoxide dismutase
PAL: Phenylalanine ammonia-lyase
CAT: Catalase
POD: Peroxidase
ROS: Reactive oxygen species
DEGs: Differentially expressed genes
RT-qPCR: Real time quantitative polymerase chain reaction
NCBI: National Center for Biotechnology Information
AP: Arg phosphate
CDS: Coding sequence
CTAB: Cetyltrimethylammonium bromide
PCR: Polymerase chain reaction
HSD: Honestly Significant Difference
