# Supplementary material for: Overexpression of OsDUF6 increases salt stress tolerance in rice
Source: BMC Plant Biol. 2024 Mar 26;24:216. doi: 10.1186/s12870-024-04921-z (PMC10964647; doi:10.1186/s12870-024-04921-z)
Supplement: Supplementary file 1 — Supplementary Material 1. [file 12870_2024_4921_MOESM1_ESM.docx]

Fig. S1. Prediction of subcellular localization of OsDUF6 protein

Fig. S2. Secondary domain analysis of OsDUF6 protein.

Fig. S3. Prediction of tertiary structure of *OsDUF6* encoded protein.

Fig. S4. PCR detection of overexpressing positive plants.

Table. S1. The list of primer sequence.

**Fig. S1.** Prediction of subcellular localization of OsDUF6 protein


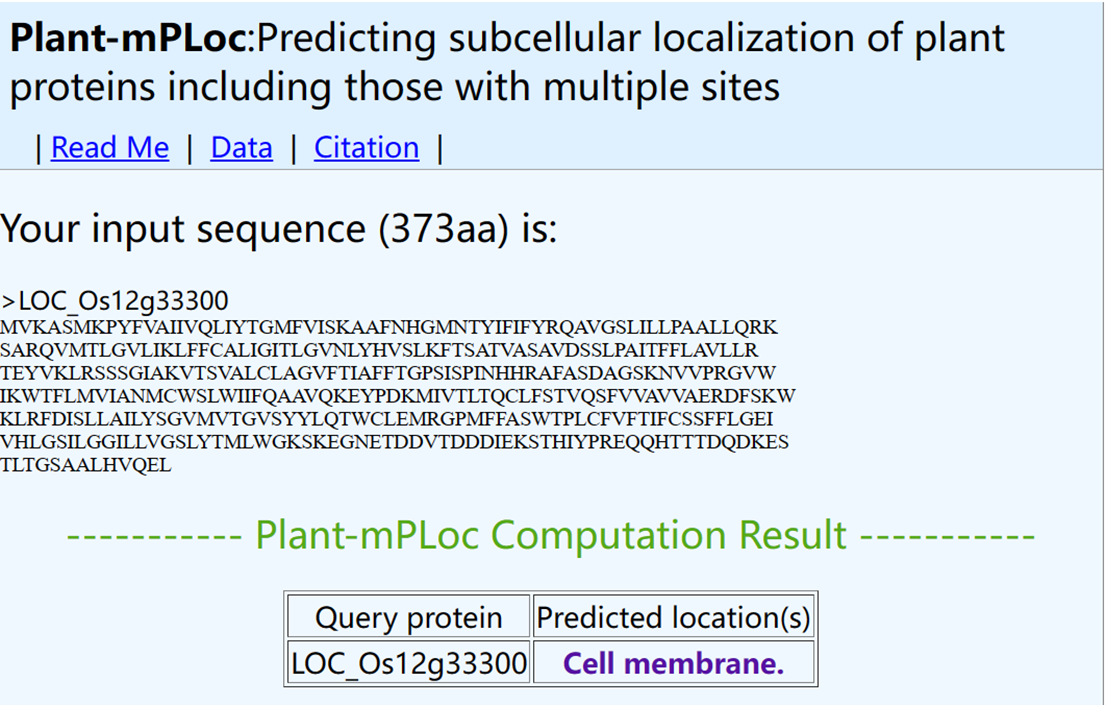


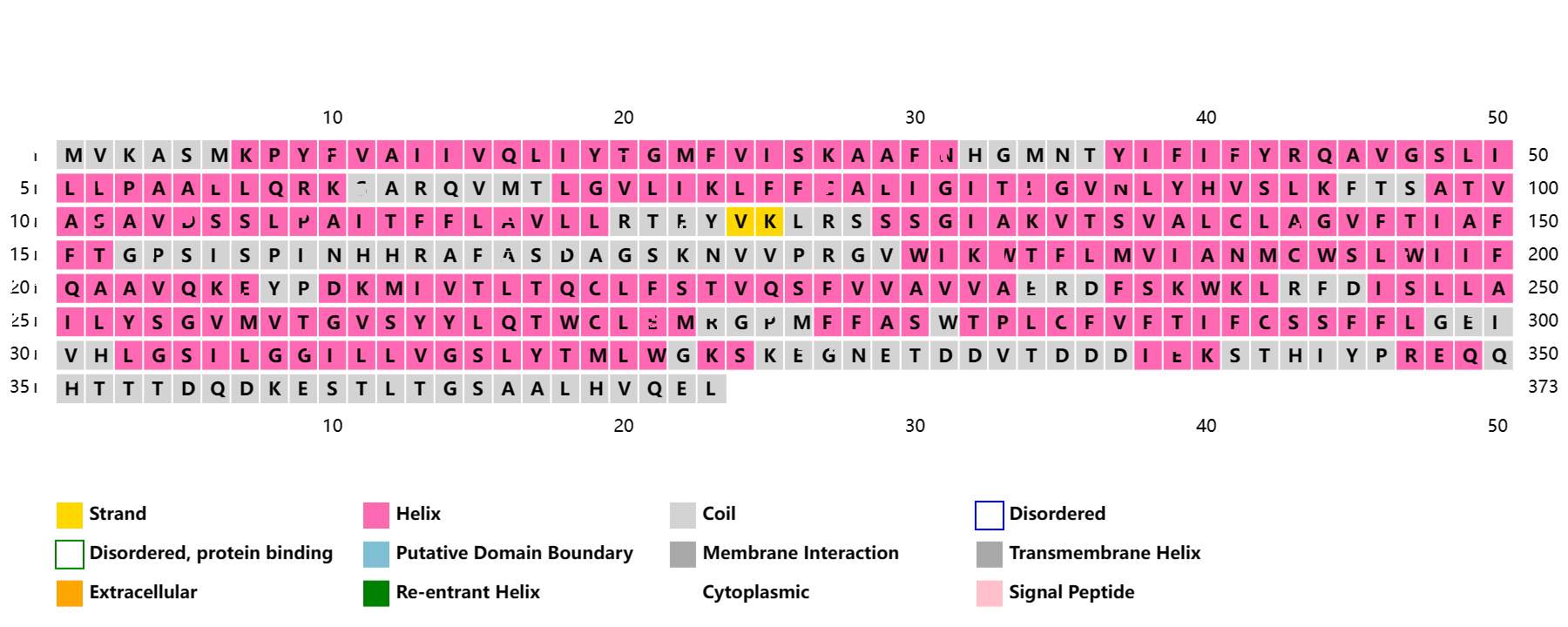
**Fig. S2.** Secondary domain analysis of OsDUF6 protein.

**Fig. S3.** Prediction of tertiary structure of OsDUF6 encoded protein. AlphaFold produces a per-residue confidence score (pLDDT) between 0 and 100. Some regions with low pLDDT may be unstructured in isolation


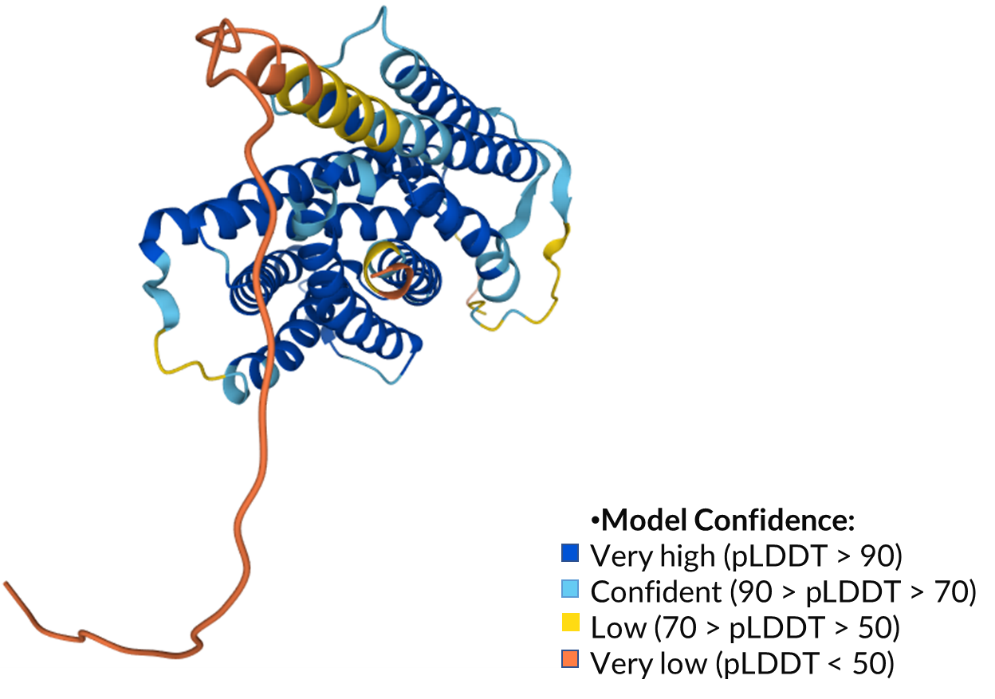


**Fig. S4.** PCR detection of overexpressing positive plants. Lane M: DNA molecular weight standard. Lanes 1-12: rice samples.


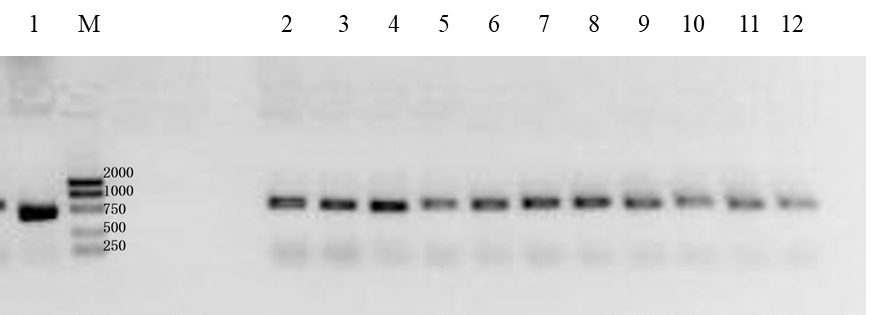


**Table. S1.** The list of primer sequence.

| Gene name | Primer sequence | Description | Product size (bp) | purpose |
| --- | --- | --- | --- | --- |
| LOC_Os11g26790 | F: CTCGTCTGAGGATGATGGAATG | dehydrin | 224 | RT-qPCR |
|  | R: TGTCCATGATGCCCTTCTTC |  |  |  |
| LOC_Os01g08320 | F: GGTGAACGGGACGGAGTA | OsIAA1 - Auxin-responsive Aux/IAA gene family member | 151 | RT-qPCR |
|  | R: GGCTCTTGGTGCTAAGTTGA |  |  |  |
| LOC_Os02g56120 | F: TTGATTTGGCCCTCCATTCC | OsIAA9 - Auxin-responsive Aux/IAA gene family member | 190 | RT-qPCR |
|  | R: AAATCATCCCATGGCACGTC |  |  |  |
| LOC_Os08g10290 | F: CAAGGTTACTACGGACCAATGA | SHR5-receptor-like kinase | 197 | RT-qPCR |
|  | R: CCACCTTCTCCCACCATATTT |  |  |  |
| LOC_Os02g42150 | F: GCCTACTCTTGGAGCAACTAAT | OsWAK14 - OsWAK receptor-like protein kinase | 166 | RT-qPCR |
|  | R: ACACGCTGGTCAGATAGAATG |  |  |  |
| LOC_Os04g51040 | F: CAAGGCTAGTCCCTTCCAATC | OsWAK50 - OsWAK receptor-like protein kinase | 189 | RT-qPCR |
|  | R: TCCCTTCCTCTTGCCTATGA |  |  |  |
| LOC_Os04g30240 | F: GCGCCTATGCTTGTCTATGAA | OsWAK60 - OsWAK receptor-like protein kinase | 194 | RT-qPCR |
|  | R: AGGATGTTGGCTGGCTTTAC |  |  |  |
| LOC_Os04g32480 | F: CTCCGATGACGCTCTTCTAC | zinc-finger protein | 163 | RT-qPCR |
|  | R: GTTCTTGTGAGTGGCTCTTTG |  |  |  |
| LOC_Os01g19800 | F: CACGTTCACGGCTGTTCT | zinc finger, C3HC4 type | 178 | RT-qPCR |
|  | R: GTATTGCCTCTGACCCTTCTG |  |  |  |
| LOC_Os04g51830 | F: GCTCTTCGTCCTCATGATGTAT | OsHKT1;4 - Na+ transporter | 186 | RT-qPCR |
|  | R: AGCTTTCTTCTCTCGGTGATG |  |  |  |
| OE-OsDUF6 | F: TTCATTTGGAGAGAACACGGGG GAC | - | 712 | overexpression identification |
|  | R: GACTGCACCGTGCTGAAC |  |  |  |
